# Exploring the Natural Variation for Seedling Traits and Their Link with Seed Dimensions in Tomato

**DOI:** 10.1371/journal.pone.0043991

**Published:** 2012-08-30

**Authors:** Noorullah Khan, Rashid H. Kazmi, Leo A. J. Willems, Adriaan W. van Heusden, Wilco Ligterink, Henk W. M. Hilhorst

**Affiliations:** 1 Wageningen Seed Lab, Laboratory of Plant Physiology, Wageningen University, Wageningen, The Netherlands; 2 Wageningen University & Reseach Centre Plant Breeding, Wageningen, The Netherlands; United States Department of Agriculture, United States of America

## Abstract

The success of germination, growth and final yield of every crop depends to a large extent on the quality of the seeds used to grow the crop. Seed quality is defined as the viability and vigor attribute of a seed that enables the emergence and establishment of normal seedlings under a wide range of environments. We attempt to dissect the mechanisms involved in the acquisition of seed quality, through a combined approach of physiology and genetics. To achieve this goal we explored the genetic variation found in a RIL population of *Solanum lycopersicum* (cv. Moneymaker) x *Solanum pimpinellifolium* through extensive phenotyping of seed and seedling traits under both normal and nutrient stress conditions and root system architecture (RSA) traits under optimal conditions. We have identified 62 major QTLs on 21 different positions for seed, seedling and RSA traits in this population. We identified QTLs that were common across both conditions, as well as specific to stress conditions. Most of the QTLs identified for seedling traits co-located with seed size and seed weight QTLs and the positive alleles were mostly contributed by the *S. lycopersicum* parent. Co-location of QTLs for different traits might suggest that the same locus has pleiotropic effects on multiple traits due to a common mechanistic basis. We show that seed weight has a strong effect on seedling vigor and these results are of great importance for the isolation of the corresponding genes and elucidation of the underlying mechanisms.

## Introduction

The success of germination, seedling establishment and later growth and development of every agricultural crop depends on many factors. Among the various factors seed quality is one of the most important to affect the success of crops [1]. High quality seed is a composite term used for all the attributes that add to the performance of a seed: genetically and physically pure, vigorous, viable, a high rate of germination, free from seed borne diseases and heat damage and produce normal seedlings under various environmental (stress) conditions [2], [3], [4]. Seed quality is also drastically affected by various environmental conditions during seed development, as well as subsequent harvesting methods, handling, and storage conditions. All these environmental factors interact with the seed’s genetic make-up [5], [6], [7].

Good seedling establishment and seedling vigor are essential for sustainable and profitable crop production and is therefore considered the most critical stage of a developing crop. Low seed vigor greatly influences both the number of emerging seedlings, and the timing and uniformity of seedling emergence. This has a major impact upon many aspects of crop production that determine cost effectiveness and the inputs required, and also has direct influence on the yield and marketing quality of a crop [1], [8] and subsequent efforts or amount of inputs during later stages of crop development will not compensate for this upshot. In tomato, huge phenotypic variation has been observed among the seeds of different species. The seeds of cultivated tomato have developed to be several times larger than their wild counterparts as a result of domestication and breeding [9]. A number of QTL studies carried out on several populations of interspecific crosses between cultivated tomato and their wild relatives have allowed the identification of loci controlling seed weight [10]–[13]. Seed weight is an indication of the reserves that seeds contain and large and heavy seeds reveal that the seed has more reserved food [14]. Many studies have shown that initial seedling size is positively related to seed size, and larger seeds have better seedling survival rate as well as higher competitiveness both within species [15]–[22] and among species [18], [20]–[31]. The seed supplies the embryo with sufficient nutrition and energy during germination from the food reserves that the seed acquires during the seed filling phase. Thus the seed filling phase plays a crucial role in successful establishment of an autotrophically growing seedling by supplying nutrition and energy and bridging the gap between germination and establishment of green cotyledons that are capable of photosynthesis [32], [33].

Root systems perform the crucial task of providing water, nutrients and physical support to the plant. The length of the main root and the density of the lateral roots determine the architecture of the root system in tomato and other dicots and play a major role in determining whether a plant will succeed in a particular environment [34]. Seed size may have an essential role in improvement of root architecture during its initial downward growth [24]. Dissecting natural variation in seed vigor of *Brassica oleracea,* [35] found a strong effect of seed vigor on the initial downward growth of seedlings and fine mapped QTLs for rapid initial growth of root which co-located with seed weight QTLs.

Little is known about the role of tomato seed size in seedling growth. In tomato, seed germination and early seedling growth are the most sensitive stages to environmental stresses such as salinity, drought and extreme temperatures [36] and most of the cultivated tomatoes are considered to be sensitive to abiotic stress conditions [37], [38], [39]. Considerable genetic variation for abiotic stress tolerance exists within cultivated tomato (*Solanum lycopersicum)*, as well as in its related wild species such as *S. habrochaitis, S. pimpinellifolium*, and *S. pennellii* [40]–[43]. The wild type tomato germplasm is a rich source of desirable genetic variability and many wild species have been identified with higher tolerance to abiotic stresses [44], [45], [46]. Among the wild species of tomato, *S. pimpinellifolium* provides numerous benefits for studying the natural genetic variation and morphological characters. It is amenable to experimental culture, readily hybridized, quick-growing, highly reproductive, relatively well known genetically and relatively resistant to biotic and abiotic stress [46]–[49] and it is closely related to *S. lycopersicum*. Despite their close relationship, the two species differ greatly in many morphological and economically interesting traits, not only in fruit size and growth traits [13], [50], but also in seed size [9], [13], [51].

In general, seed and seedling vigor characteristics are complex traits, which are probably controlled by several genes and are therefore suitable for quantitative trait loci (QTL) analysis. In the current study we analysed these traits in a recombinant inbred line (RIL) population between *S. lycopersicum* (cv. Money maker) and *S. pimpinellifolium* [52], [53]. The study revealed the presence of high phenotypic variability in the population with regard to seed size, seedling growth and root architecture and due to this variability we were able to identify 62 QTLs related to seed and seedling traits. In addition the results also revealed a strong correlation between seed size and seedling growth and co-location of QTLs for these traits.

## Materials and Methods

### Plant Material

The tomato RIL population was obtained from a cross between *Solanum lycopersicum* cv. Moneymaker and *Solanum pimpinellifolium* CGN 15528 [52]. This population was genotyped for a total of 865 Single Nucleotide Polymorphism (SNP) markers in F_7_ and produced 83 RILs in the F_8_. The genotyping was done with a custom made, in house SNP array based on polymorphisms detected with 454 (Roche) and Illumina sequencing in 8 different tomato species (personal communication AW van Heusden).

### Growth Conditions and Seed Collection

The RIL population of *S. lycopersicum* X *S. pimpinellifolium* was grown twice under controlled conditions in the greenhouse facilities at Wageningen University, the Netherlands. The day and night temperatures were maintained at 25 and 15°C, respectively, with 16 h light and 8 h dark (long-day conditions). All the RILs were uniformly supplied with the basic dose of fertilizer.

Seeds were collected from healthy mature fruits and subsequently treated with 1% hydrochloric acid (HCL) for 1.5 h to remove the pulp sticking onto the seeds. The solution of tomato seed extract with diluted hydrochloric acid was passed through a fine mesh sieve and washed with tap water to remove pulp and hydrochloric acid. The seeds were processed and disinfected by soaking in a solution of trisodium phosphate (Na3PO4.12H2O). Finally, seeds were dried on filter paper at room temperature and were brushed to remove impurities with a seed brusher (Seed Processing Holland BV, Enkhuizen, The Netherlands, http://www.seedprocessing.nl). The cleaned seeds were dried for 3 d at 20°C and stored in a storage room (13°C and 30% RH) in paper bags. The seeds of each harvest were bulked separately for each RIL and were used in the subsequent experiments.

### Linkage Analysis

The genetic linkage map consists of 12 individual linkage groups corresponding to the 12 chromosomes of tomato and was made on the basis of genotyping the segregation of parental alleles in the *S. lycopersicum* cv. Moneymaker X *S. pimpinellifolium* G1.1554 RIL population with 865 SNP markers. See Kazmi *et al.* [53] for more details.

### Phenotyping of Seed Traits of the RIL Population

Seed weight (SW) was measured as the average seed weight of a batch of 100 seeds. Seed size was determined by taking close-up photographs from 2×100 seeds using a Nikon D80 camera with a 60 mm objective fixed to a repro stand and connected to a computer, using Nikon camera control pro software version 2.0 [54]. The photographs were analyzed using the open source image analysis suite ImageJ (http://rsbweb.nih.gov/ij/) by using color-thresholds combined with particle analysis that automatically scored seed size (SS) as the area of selection in square pixels, circularity (SC) as 4π*(area/perimeter^2^) and seed length (SL) as the longest distance between any two points along the selection boundary (feret’s diameter). Seed size and seed length were also determined in 12-h imbibed seeds (ImbSS and ImbSL, respectively).

### Seedling Growth

Seedling growth was tested in three independent experiments. In the first two experiments seedlings were grown on vertical plates (12×12 cm square Petri dishes) on half MS medium under aseptic conditions at pH 5.6. The top 4 cm of the agar solution was removed with a sterilized knife and the seedlings were grown on the remaining 8 cm. In each experiment 7 seedlings were grown per plate in a randomized complete block design for each harvest in duplicate (7*2*2 seedlings per experiment) in a climate chamber at 25°C with long day conditions (16 h light, 8 h dark). Before sowing, seeds were surface sterilized for 16 h in a desiccator over a solution of 100 ml 4% sodium hypochlorite +3 ml concentrated hydrochloric acid.

Germination was scored at 8-h intervals as visible radical protrusion. After the start of germination photographs were taken at 24–h intervals for root architecture analysis. Five days after germination the hypocotyl length and the fresh root and shoot weight data were measured (HypL, FrRt and FrSh respectively). After subsequent drying for 1 week at 90°C the dry root and shoot weights were measured (DrRt and DrSh respectively). Root system architecture was analyzed with the EZ-Rhizo software package [55] to obtain parameters such as total root size (TRS), main root length after five days (MRL), number of lateral roots per main root (LRn) and lateral root density per branch zone (LRD-Bz).

In a third experiment seedlings were grown under nutrient-deprived conditions on a Copenhagen table. The seedlings were grown on blue filter paper and were covered with conical glasses with a small hole on the top. These conical glasses prevent the loss of moisture provided by the Copenhagen table without blocking aeration of the seedlings. Each harvest was tested separately in two consecutive sub-sets of experiments. Twenty seeds of each RIL for each seed harvest were germinated on Copenhagen tables in a randomized complete block design in triplicate (20×3×2 harvests). Germination was recorded as visible radical protrusion at 8-h intervals. The first 10 germinated seeds were allowed to develop into a seedling and ten days after reaching the t_50_ (time to 50 percent germination) the seedlings were harvested and the fresh and dry root and shoot weight data were determined (FrRtwn, DrRtwn, FrShwn and DrShwn, respectively). In this case we could not assess the root architecture due to the set-up of the Copenhagen table on which the roots grow horizontally and become intertwined.

### Data Analysis

Pearson correlations between different traits were calculated with the PASW statistics software, version 17 [56]. QTL analyses was performed with the mapping software MapQTL®5.0 [57]. In a first step, putative QTLs were identified using interval mapping. Thereafter, the estimated additive effect and the percentage variance explained by each QTL, as well as the total variance explained by all of the QTLs affecting a trait, were obtained by MQM mapping. For this purpose different markers were tested around a putative QTL position as a cofactor (Van Ooijen and Maliepaard, 1996) and those maximizing the LOD score were selected as the final cofactors and finally restricted multiple QTL mapping (rMQM) was used to obtain the confidence intervals. A LOD score of 2 was calculated as a threshold level with a permutation test to detect statistically significant QTL.

### Analysis of Heritability and Epistasis

Broad-sense heritability (h^2^_b_) was estimated from one-way random-effects of analysis of the variance (ANOVA, SPSS version 19.0) with the equation: h^2^_b_ = σ^2^_g_/(σ^2^_g_ + σ^2^_e_) where σ^2^_g_ is the genetic variance and σ^2^_e_ is the environmental variance [58]. Significant differences among all means of the RILs were estimated using one-way ANOVA followed by a least significant difference (LSD) test.

A two-dimensional genome-wide epistatic interactions analysis was performed using the R/qtl software package [59] in order to identify epistatic interactions contributing to variation in traits. This includes nested linear model-fitting for each pair of loci [60]. Genome-wide significance thresholds were obtained by 10,000 permutation tests [61] with the Haley-Knott regression method [59]. LOD significance threshold of the maximum genome-wide interaction (lod.int), full model (lod.full), and conditional interactive model (lod.fv) were found to be 4.09, 6.04 and 4.63, respectively.

## Results

### Phenotypic Variation in Seed and Seedling Vigor Related Traits

In total 19 traits were tested in this study, including 6 seed traits, such as seed weight (SW), seed size (SS), seed length (SL), seed circularity (SC), imbibed seed size (ImbSS) imbibed seed length (ImbSL) and 5 seedling- and 4 root architecture related traits. The seedling related traits included fresh and dry root and shoot weight (FrRt, DrRt, FrSh and DrSh respectively), and hypocotyl length (HypL). The 4 root architecture related traits, included main root path length (MRL), total root size (TRS), lateral root number (LRn), and lateral root density per branched zone (LRD/Bz) in both experiments. Differences between the two parents were statistically highly significant for all the traits studied (P<0.01 to 0.001) with the *S. lycopersicum* parent having higher trait values as compared to the *S. pimpinellifolium* parent in all the traits except LRD/Bz (Table 1). In addition, there were statistically significant differences for these traits among the different lines of the RIL population (Table 1).

**Table 1:** Phenotypic analysis of seed and seedling related vigor traits of a *S. lycopersicum* and *S. pimpinellifolium* RIL population and its two parents.

Nr	Trait^1^	S. lycopersicum	S. pimpinellifolium	RIL Population	F-Value^3^	P-Value^3^
		Mean	Mean	Mean±SD^2^		
1	FrRt	20.30	10.90	15.91±5.21	3.58	0.001
2	DrRt	1.97	0.56	1.19±0.36	2.13	0.001
3	FrSh	46.27	17.01	32.47±8.97	4.51	0.001
4	DrSh	3.04	1.18	2.18±0.50	4.50	0.001
5	HypL	3.20	2.08	2.83±0.61	4.00	0.001
6	SW	2.95	1.08	1.70±0.38	2.76	0.001
7	SS	4.40	2.34	3.26±0.50	16.35	0.001
8	SL	2.93	1.62	2.51±0.21	1.56	0.012
9	ImbSS	6.45	3.42	4.72±0.75	14.52	0.001
10	ImSL	3.79	2.01	3.08±0.25	1.39	0.046
11	FrShwn	27.20	7.28	13.37±3.54	8.27	0.001
12	DrShwn	1.47	0.37	0.77±0.20	7.20	0.0001
13	FrRtwn	14.64	5.48	9.06±2.52	10.89	0.001
14	DrRtwn	0.95	0.31	0.52±0.15	2.96	0.001
15	MRL	8.54	4.61	6.93±1.18	3.47	0.001
16	TRS	13.99	6.36	10.18±2.38	3.53	0.001
17	LRn	8.60	3.86	4.65±2.15	3.57	0.001
18	LRD/BZ	3.41	6.08	4.65±2.90	1.15	0.245

1 FrRt = Fresh Root weight, FrSh = Fresh shoot weight, DrRt = Dry root weight. DrSh = Dry Shoot weight, HypL = Hypocotyl length, SW = Dry Seed weight. SS = Dry seed size, SL = Dry seed length, SC = Dry Seed circularity, ImbSS = imbibed seed size, ImbSL = Imbibed seed length, FrShwn = Fresh Shoot weight under nutrientless condition, DrShwn = Dry shoot weight in nutrientless condition, FrRtwn = Fresh root weight in nutrientless condition, DrRtwn = Dry root weight under nutreintless condition, MRL = Main Root path Length, TRS = Total root size, LRn = Lateral root number per main root, LRD/Bz = Lateral roots density per branched zone.

2 standard deviation.

3 F-value and P- value were calculated for the population mean.

**Figure 1:**
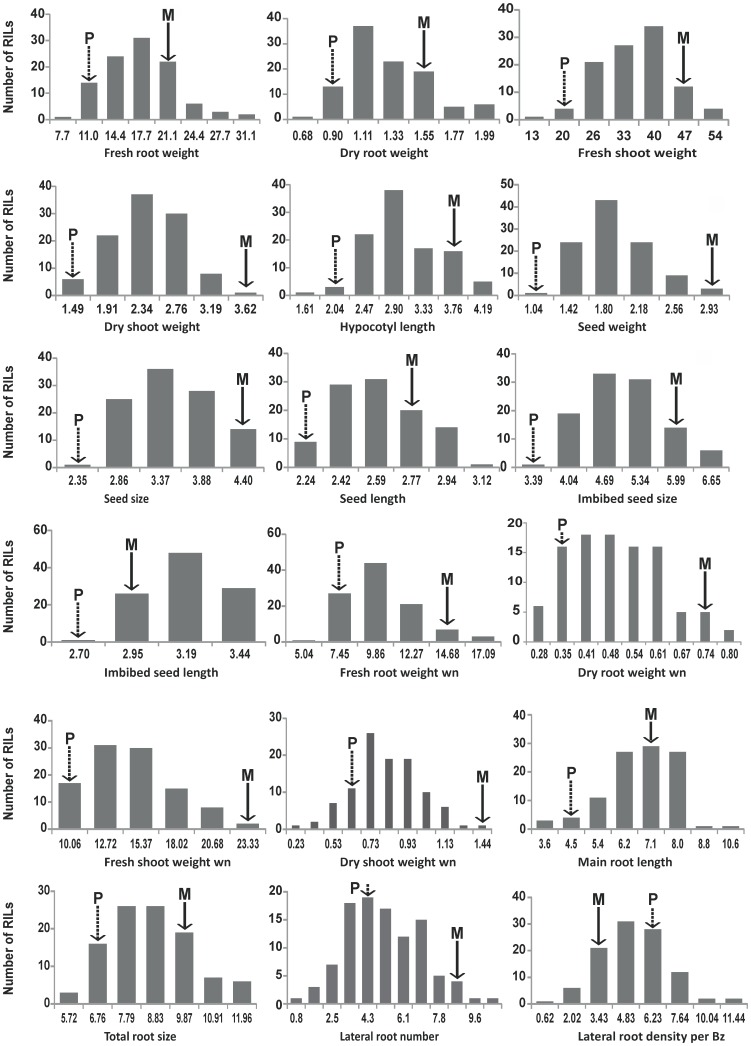
Frequency distributions of non-normalized data of all measured seed and seedling phenotypes in the *Solanum lycopersicum* x *Solanum pimpinellifolium* RIL population. wn: without nutrition. The parental values are indicated with a solid arrow. P = *S. pimpinellifolium* parent and M = *S. lycopersicum* parent.

Besides testing on agar plates, we measured seedling growth of the RIL population also on a Copenhagen table without any nutrition, to test the importance of amount of reserve food present in the seed (seed vigor) in the form of total biomass acquired by the seedling in a specific period of time from radical protrusion until harvesting of the seedling. In this experiment we measured fresh and dry root and shoot weight (FrRtwn, DrRtwn, FrShwn and DrShwn respectively). We observed significant differences between the two parents as well as in the RIL population for the seedling traits measured during this experiment (Table 1). In addition we also observed significant differences between the seedling traits tested across the two experiments. There was 27 to 56% decrease in the biomass gained in ten days after germination under the nutrientless condition as compared to the mass obtained in five days after germination under the normal nutrient conditions (Table 2). All measured traits showed a normal distribution over the RIL population (Fig. 1). Figure 1 also shows that transgression was present for most traits.

**Table 2:** Reduction in biomass of seedling grown under nutrient stress condition as compared to the biomass obtained under normal nutrient conditions.

Normal^1^				Wn^2^				Decr^3^		
Trait^4^	*S.lyco*	*S.pimp*	RILs	Trait^5^	*S.lyco*	*S.pimp*	RILs	*S.lyco*	*S.pimp*	RILs
	Mean	Mean	Mean		Mean	Mean	Mean			
FrRt	20.30	10.90	15.90	FrRtwn	14.64	5.48	9.06	27.9%	49.7%	43.1%
DrRt	1.97	0.56	1.19	DrRtwn	0.95	0.31	0.52	51.8%	44.6%	56.3%
FrSh	46.27	17.01	32.50	FrShwn	27.21	7.28	13.37	41.2%	57.2%	58.8%
DrSh	3.04	1.18	2.18	DrShwn	1.47	0.37	0.77	51.6%	68.7%	64.7%

1 Normal = Seedling grown under normal nutrients condition,

2 Wn = Seedling grown on Copenhagen table without nutrition,

3 Decr = Seedling grown on Copenhagen table without nutrition,

4 FrRt = Fresh Root weight, DrRt = Dry root weight, FrSh = Fresh shoot weight, DrSh = Dry Shoot weight,

5 FrRtwn = Fresh root weight in nutrientless condition, DrRtwn = Dry root weight under nutrientless condition, FrShwn = Fresh Shoot weight under nutrientless condition, DrShwn = Dry shoot weight in nutrientless condition.

### Correlation between Traits

**Figure 2:**
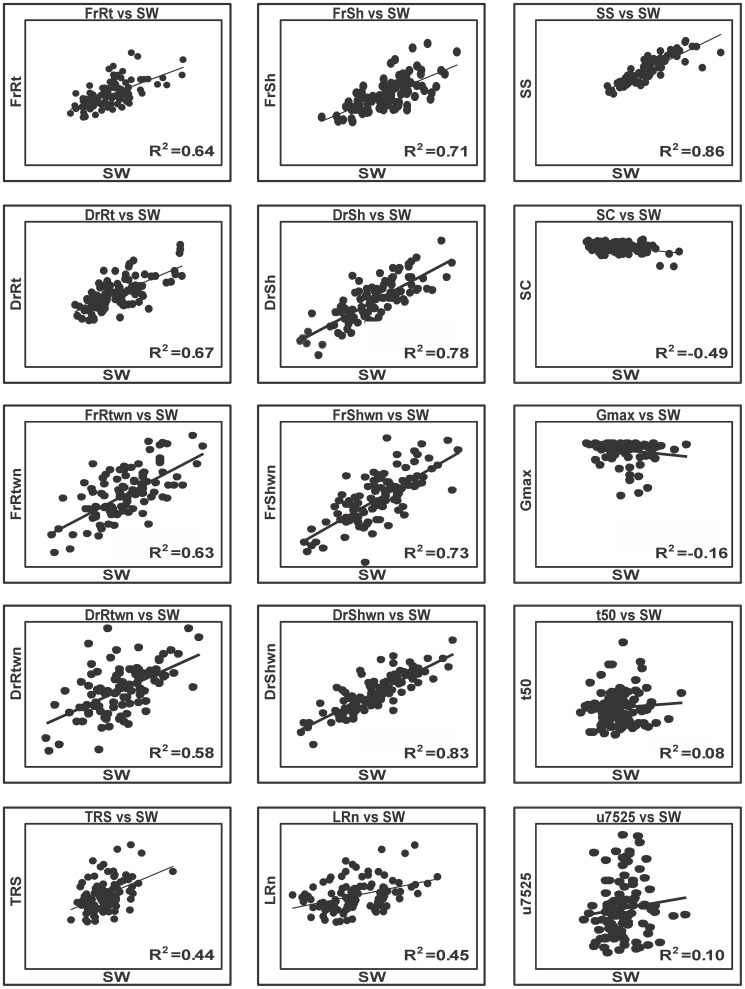
Correlation among seed and seedling traits. SW = Seed weight, SS = Seed size SL = Seed length. FrRt = Fresh root weight, DrRt = Dry root weight, FrSh = Fresh shoot weight, DrSh = Dry shoot weight, FrShwn = Fresh shoot weight in nutrientless conditions, DrShwn = Dry shoot weight in nutrientless conditions, FrRtwn = Fresh root weight in nutrientless conditions, DrRtwn = Dry Root weight in nutrientless conditions, G_max_ = Maximum total germination in %, t_50_ = time to complete 50% germination, U_7525_ = Uniformity of germination (time between 25 to 75% germination).

Statistically significant correlations were observed between seed weight and seedling traits such as fresh and dry root and shoot weight (Fig. 2). The R^2^ value for the Pearson correlation between seed weight and different seedling traits varied from 0.64 for seed weight vs. fresh root weight to 0.78 for seed weight vs. dry shoot weight (Fig. 2). Under the nutrient-deprived condition the R^2^ value varied from 0.58 to 0.83 between seed weight and dry root and shoot weight (DrRtwn and DrShwn). In addition, we found statistically significant correlations among seed traits such as seed size and seed length and seedling traits,as expected (data not shown). On the other hand, although we found we found significantly negative correlation between seed size and seed circularity, we found no correlations between seed circularity and seedling traits. In case of root architecture, we found low (R^2^ value 0.44 and 0.45), but statistically highly significant (p value 0.001) correlations between seed weight and total root size (TRS) and lateral root number(LRn), but could not find any correlation with the other root traits (MRL and LRD/Bz). (Fig. 2). We also tested the correlation between seed traits and seed performance such as total germination percentage (G_max_%), rate of germination (t_50_) and uniformity of germination (U_7525_) [53], but found no significant correlations between seed traits and seed germination parameters, which is obvious from the R^2^ values (Fig. 2).

### Mapping QTLs for Different Traits

**Figure 3:**
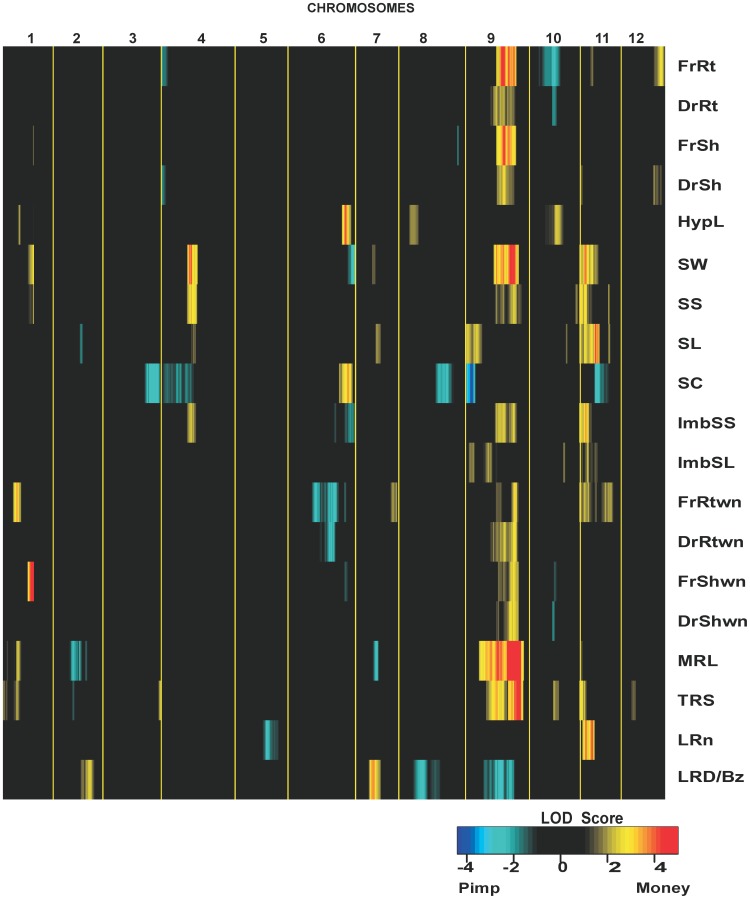
Heatmap of QTLs identified for seed and seedling quality traits. Tomato chromosomes are identified by numbers (1–12), with centimorgans ascending from the left to right; chromosomes are separated by yellow lines. SW = Seed Weight, SS = Seed Size SL = Seed Length. FrRt = Fresh Root weight, DrRt = Dry Root weight, FrSh = Fresh Shoot weight, DrSh Dry Shoot weight, FrShwn = Fresh Shoot weight in nutrientless conditions, DrShWn = Dry Shoot weight in nutrientless conditions, FrRtwn = Fresh Root weight in nutrientless conditions, DrRtwn = Dry Root weight in nutrientless conditions, MRL = Main Root Length, TRS = Total Root Size, LRn = Lateral Root number per main root, LRD/Bz = Lateral Root Density per Branched zone. Colors indicate QTLs significant at *P* = 0.002 in multiple QTL mapping models (1-LOD intervals). Blue and light blue colors indicate a larger effect of the trait in *S. pimpinellifolium*, and yellow and red in *S. lycopersicum*.

We used the data of the studied seed, seedling and RSA phenotypes under control and nutrient-deprived conditions to map QTLs with the use of a LOD threshold of 2.0. Multiple QTL (MQM) mapping analysis revealed a total of 62 significant QTLs on 21 different positions for the 19 seed and seedling traits tested across the RIL population (Table 3). By making a heat map of LOD profiles, QTLs can be visualized and global ‘hot spots’ and empty regions across the 12 chromosomes can be seen (Fig. 3). Co-localization of QTLs was found for different seed and seedling traits on the bottom of chromosomes 1, 4, 6, 9 and 11 (Table 3, Fig. 3). Out of the 62 detected QTLs, 25 were related to seed traits, such as seed weight, seed size, seed length and seed circularity. Seventeen QTLs were related to seedlings biomass, such as fresh and dry root and shoot weight (across both the growing conditions) and 3 QTLs to hypocotyl length, whereas 17 QTLs were related to root system architecture. We identified significant QTLs for all the traits, ranging from 1 to 6 QTLs per trait with LOD scores in the range of 2.1 to 6.4. Explained variances for single QTL ranged from 4.8% for the QTL for total root size on chromosome 10 to 24.6% for the QTL on chromosome 1 for fresh shoot weight without nutrition. The total explained variance for different traits caused by these QTLs varied from 11.9% for dry root weight to 62.9% for seed weight with genetic heritability ranging from 0.53 for lateral root density to 0.94 for seed size. About 72.5% of the favorable alleles were derived from the *S. lycopersicum* parent (negative additive effects in Table 3).

**Table 3:** Overview of significant QTLs associated with seed and seedling traits of *S. lycopersicum* and *S. pimpinellifolium* tomato RIL population.

Trait^1^	Chr^2^	Confidence Interval (cM)	Nearest Marker^3^	LODscore	AdditiveEffect^4^	Explained Variance (%)	Total Exp Variance (%)	Heritability
**FrRt**								
	9	54.8–91.8	62162316	3.3	−0.73	14.1	30.8	0.78
	10	8.6–100.4	58738936	2.1	0.59	8.5		
	12	0–79.8	62040100	2.0	−0.55	8.2		
**DrRt**								
	9	46.7–101.1	60488088	2.6	−0.70	11.9	11.9	0.68
**FrSh**								
	9	59.0–96.3	62897108	3.4	−0.78	16.0	16.0	0.82
**DrSh**								
	4	0–20.9	30398	2.6	0.63	9.7	25.1	0.82
	9	65.0–88.5	62897108	3.4	−0.75	15.4		
**HypL**								
	1	18.9–64.9	2766897	2.0	−0.54	7.4	33.7	0.80
	6	87.3–99.2	41812268	4.2	−0.84	17.0		
	10	1.6–80.2	59476312	2.4	−0.69	9.3		
**SW**								
	1	49.9–64.9	69227784	3.1	−0.56	8.6	60.9	0.73
	4	50.4–63.8	51677496	4.6	−0.69	13.5		
	6	95.8–109.3	44905196	3.1	0.57	8.5		
	9	54.8–95.3	60488088	4.2	−0.68	12.1		
	9	54.2–94.3	64960580	3.6	−0.63	8.4		
	11	0–28.5	4775141	3.7	−0.62	9.8		
**SS**								
	1	44.8–64.9	69430752	2.2	−0.49	7.0	36.5	0.94
	4	49.4–67.7	51677496	3.7	−0.64	12.1		
	9	52.3–104.1	64960580	2.6	−0.53	8.2		
	11	0–20.6	5148394	2.9	−0.56	9.2		
**SL**								
	2	0–92.3	39990428	3.2	0.83	9.1	33.3	0.61
	9	0–35.8	48774	2.4	−0.56	8.0		
	11	22.1–33.5	48283252	4.6	−0.73	16.2		
**SC**								
	3	85.7–135.2	58802824	3.0	0.64	8.1	51.9	0.70
	4	0–74.1	3902301	2.0	0.50	5.4		
	6	86.3104.3	42299156	3.9	−0.70	11.1		
	8	79.3–124.4	57594496	2.6	0.56	7.0		
	9	0–16.7	1751657	4.4	0.75	12.6		
	11	20.6–52.1	48283252	2.8	0.57	7.7		
**ImbSS**								
	4	46.0–69.2	51677496	2.6	−0.59	9.3	41.3	0.93
	6	58.5–109.3	43431568	2.2	0.53	7.6		
	9	56.0–93.0	64960580	3.0	−0.65	10.9		
	11	0–16.0	5148394	3.7	−0.72	13.5		
**ImbSL**								
	9	28.5–63.5	5400867	2.6	−0.68	10.6	21.3	0.58
	11	0–36.4	5472482	2.3	−0.65	10.7		
**FrRtwn**								
	1	20.5–36.3	2746777	3.6	−0.66	11.7	45.2	0.89
	6	36.6–81.6	39180864	3.0	0.59	9.5		
	7	64.3–90.7	61282892	2.0	−0.48	6.5		
	9	81.3–95.3	64960580	3.1	−0.60	10.0		
	11	0–68.4	4775141	2.4	−0.52	7.5		
**DrRtwn**								
	6	43.6–80.5	37874180	2.1	0.64	9.9	23.6	0.88
	9	46.7–95.3	62897108	2.9	−0.71	13.7		
**FrShwn**								
	1	57.9–64.9	69430752	6.4	−1.01	24.6	36.1	0.92
	9	76.4–96.3	64960580	3.3	−0.69	11.5		
**DrShwn**								
	9	70.3–96.3	64960580	3.2	−0.78	14.6	14.6	0.75
**MRL**								
	1	1–39.5	2746777	2.6	−0.51	6.1	41.3	0.65
	2	29.4–67.8	37722740	2.5	0.59	6.0		
	7	33.2–55.3	28075704	2.7	0.53	6.5		
	9	26.4–104.7	62162316	3.5	−0.63	8.5		
	9	76.4–98.8	65815200	5.7	−0.87	14.2		
**TRS**								
	1	0–39.5	2746777	2.1	−0.49	5.6	51.4	0.79
	3	59.7–135.2	61881752	2.2	−0.53	5.9		
	9	39.4–75.1	60488088	4.1	−0.70	11.3		
	9	77.4–101.1	65815200	5.6	−0.86	15.7		
	10	9.3–82.2	58738936	2.1	−0.46	4.8		
	11	0–12.1	4106782	3.0	−0.60	8.1		
**LRn**								
	5	53.4–86.1	6814273	2.9	0.71	13.0	32.1	0.78
	11	2.4–22.7	5148394	4.1	−0.87	19.1		
**LRD/Bz**								
	2	50.0–83.8	43635344	2.6	−0.70	9.4	44.9	0.53
	7	29.2–56.3	3317484	3.8	−0.81	14.5		
	8	22.2–98.9	2908496	2.5	0.64	9.3		
	9	33.8–88.7	62162316	3.2	0.69	11.7		

1 FrRt = Fresh Root weight, DrRt = Dry root weight, FrSh = Fresh shoot weight, DrSh = Dry Shoot weight, HypL = Hypocotyl length, SW = Dry Seed weight. SS = Dry seed size, SL = Dry seed length, SC = Dry Seed circularity, ImbSS = Imbibed seed size, ImbSL = Imbibed seed length, FrShwn = Fresh Shoot weight under nutrientless condition, FrRtwn = Fresh Root weight under nutrientless condition, DrShwn = Dry shoot weight under nutrientless condition, DrRtwn = Dry root weight under nutrientless condition, MRL = Main Root Length, TRS = Total root size, LRn = Lateral root number per main root, LRD/Bz = Lateral roots density per branched zone.

2 Chromosome on which the QTLs were detected.

3 Nearest marker to the position of the identified QTLs.

4 A positive sign means that the allele of *S. pimpinellifolium* contributed to the increase of particular trait while the negative sign means that the allele of *S. lycopersicum* increased the trait at this particular locus.

### Stress Specific QTLs

We identified QTLs that were either common across both the conditions or specific to a particular condition. For example the QTLs on chromosome 9 could be identified for the 4 seedling traits tested across both the conditions while the QTLs on chromosomes 4 and 12 for FrRt could only be identified under normal nutrient conditions (Table 3, Figure 3). On the other hand the QTLs on Chromosome 1 for FrRtwn, and FrShwn and on Chromosome 6 for FrRtwn and DrRtwn, as well as on chromosome 7 and 11 for FrRtwn were only identified under nutrient-deprived conditions.

### Epistatic Interactions

**Figure 4:**
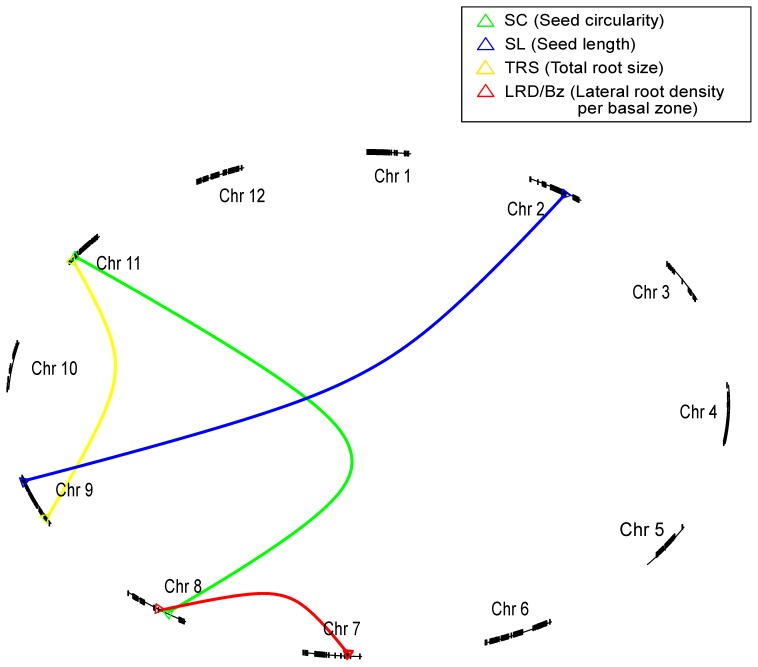
Epistatic interaction network of QTLs identified for seed and seedling quality traits. Graphical visualization of the epistatic interactions observed among different loci controlling seed and seedling quality phenotypes. The 12 chromosomes are represented as different circle segments, and their sizes are proportional to the corresponding genetic sizes measured in cM. The colour of the lines indicates the trait for which the epistatic interaction was observed [86].

For each of the described traits, a genome-wide epistasis analysis was performed. In this analysis all pairwise combinations of the markers closest to each target QTL was tested. With this method several instances of epistatic interactions among seed size and seedling QTLs were revealed (Table 4, Fig. 4). These epistatic interactions contribute to phenotypic variability, but hinder detection and affect estimation of QTLs examined singly. This analysis revealed novel loci on several chromosomes interacting to influence seed size and seedling traits. The analysis revealed loci on chromosomes 8 and 11 interacting to influence seed circularity (Table 4, Fig. 4). Similarly, for seed length, evidence of interaction was observed on chromosomes 4 and 7. A two-way interaction was also revealed for total root size on chromosomes 9 and 11. Finally, a strong interaction was observed for lateral root density between a locus on chromosome 7 and 8 (LODint = 6.97) (Table 4, Fig. 4), which had the highest level of statistical significance obtained in our epistasis screen.

**Table 4:** Interaction LOD scores for phenotypes significant at the genome-wide level (P<0.05).

Phenotype	Chr A	Position (cM)	Chr B	Position (cM)	Lod.full^a^	Lod.fv1^b^	Lod.int^c^
C	8	9	11	29	11.62	8.81	4.62
SL	2	60	9	5	7.45	5.87	4.25
TRS	9	97	11	6	13.00	8.02	6.49
LRD/Bz	7	57	8	81	9.26	7.75	6.97
SW	1	30	6	54	7.98	5.68	3.78
SW	6	54	9	87	8.49	6.20	3.77
SS	9	89	11	3	9.13	6.26	3.86

Two-way epistatic interactions for *S. lycopersicum/S. pimpinellifolium* RIL population across all 12 chromosomes.

a Lod.full is the LOD score of the full model with two loci and their interaction compared to the null model with no QTL.

b Lod.fv1 is the LOD score of the full model compared to the best single QTL model with one locus on either chromosome A or B.

c Lod.int is the LOD score of the interaction term which is found by comparing the full model with an interaction term, to the two QTL models with no interaction term.

## Discussion

During our study we found considerable variation between the two parents for all the physiological parameters tested and an even higher variation was found in the RIL population, since transgression was observed for most of the traits. The phenotypic variation in the two parents, as well as in the RIL population and the resolution and size of this population was sufficient to find QTLs for seed and seedling quality, showing that this RIL population is a powerful tool for the study of the quantitative traits under study. We have utilized homogenous and strictly controlled plant growth conditions and seedling phenotype testing and this has contributed to the high genetic heritability that we observed for most of the traits. It furthermore indicates that the measured traits have a strong genetic regulation.

In a previous study [53] we analyzed 42 seed quality traits and identified 120 QTLs under optimal and stress conditions. Thus this population provides a valuable source for exploring the genes influencing complex phenotypes for seed quality as they allow isolation of the effect of a specific QTL from those of the entire genome and consequently enhance the statistical power to unravel quantitative seed quality phenotypes, controlling complex underlying mechanisms.

The seedling’s ability for shoot penetration through the impeding soil of the seed bed is an essential attribute of vigor [62]. Rapid germination and subsequent seedling growth are, therefore, key phenotypes of vigorous seeds that are known to differ with genetic background [63]. Thus, a vigorous seed must possess three key traits to establish seedlings across a wide range of environments: (1) the seed must germinate rapidly; (2) should have rapid initial downward growth; and (3) must have high potential for rapid upward shoot growth. Data obtained from fresh and dry root and shoot weights are good indicators for estimating the downward growth rate of root and upward growth rate of shoot, as well as predicting seed vigor [63], [64], [65].

Keeping in view the background and importance of seedling vigor through testing root and shoot growth of the seedling, we analyzed our RIL population for these traits and detected 10 QTLs for seedling growth on agar plates and 10 QTLs for growth of seedlings without nutrition. In addition, we identified 17 QTLs for seedling root architecture and 25 QTLs for seed dimension related traits. Most traits were enhanced by an allele of the *S. lycopersicum* parent, which displays vigorous seedling growth and high seed weight. However 27.5% of the detected QTLs had allelic effects enhanced by the *S. pimpinellifolium* parent, but these included QTLs for SC and LRD/Bz which indicates that small seeds have higher values for seed circularity and more lateral roots per basal zone in this population. Similar results were obtained in other tomato populations with the majority of the enhancing alleles for seed weight, fruit weight and total yield [13], and different botanical traits [66] coming from the *S. lycopersicum* parent. Our results are also supported by results in other crops in which QTLs were mainly affected by the positive allele of the parent with the heavy-weighted seed, for example in a study of the root architecture in melon [65]. Besides the observed strong positive correlation between seed dimensions and seedling traits, we also found co-location of QTLs for these traits, as might be expected from these results. Co-location of QTLs for different traits can be an indication that a locus has a pleiotropic effect on multiple traits, due to a common mechanistic basis or a dependency of traits [67]. For example, a QTL on linkage group 9 is shared by five traits such as FrRt, DrRt, FrSh, DrSh and SW whereas the QTLs on linkage group 1 at marker position 69430752 are common between FrRtwn, SW and SS, respectively. In the present study most of the QTLs with major effect on all five seedling traits were identified on linkage groups 1, 6, 9 and 11. Most of these QTLs were co-locating with the QTLs for seed traits that we have identified in the current study and the QTLs identified in other studies of tomato seed weight [9], [10], [11], [12], [13]. These results are in agreement with those reported by Nieuwhof *et al.* [68], who tested 15 tomato genotypes with different seed size and 105 F1 obtained by di-allel crossing and found that genotypes with large seeds produced heavier seedlings than genotypes with small seeds. They also found a correlation between seed and seedling weight in the same range (R^2^ = 0.8) as we have found in our study. The effect of seed weight on seedling growth may be due to the genetic variation in the amount of reserve food in the seeds and possible influenced by the maternal environment during seed development and maturation.

We found no significant correlations between seed size or seed weight and seed performance, such as rate and uniformity of germination or maximum germination percentage [53], as was also found in other species [69]. Thus, seed size is beneficial to the establishment of seedlings, but there appears to be no consistent link between seed size and germination characteristics.

Many selective factors affect seed size [69]–[75]. The environment has great influence on seed size, with many factors that interact to affect the trait, such as high temperatures, short days, red light, drought and high nitrogen levels [69]. In tomato several studies have been carried out to identify QTLs for seed weight with seven different populations involving interspecific crosses between cultivated tomato and five wild tomato species [10]–[13]. The number of QTLs varied from 3 to 14 per study depending on the analytical method and the genetic populations used. In total 24 seed weight QTLs have been identified by different studies [9]. Twelve seed weight QTLs were detected in only one species while 11 seed weight QTLs in two or more different species. One of the QTLs (*sw4.1*; [76] was common among all species and we found a QTL at the same position. In spite of the large number of QTLs identified for seed weight, no attention has been given in the previous studies to seed dimensions such as seed size and seed length. Although seed size, length and seed weight are closely related traits and are interdependent on each other, we measured differences in the total number of QTLs identified for seed weight (6 QTLs), seed size (4 QTLs) and seed length (3 QTLs)(Table 3), as well as in the individual and total explained variance of QTLs for seed weight (total exp. variance 60.9%), seed size (36.5%) and seed length (33.3%). The detected QTLs for seed size are co-locating with the seed weight QTLs, but 2 of the 3 seed length QTLs are found on different locations. This indicates that although a strong correlation can be expected between the different seed dimension parameters, there are at least different loci influencing seed length as compared to seed size and weight.

A large number of QTLs for seed weight has also been identified in other crops. As an example, Teng *et al.* [77] found 94 QTLs for seed weight in soybean at different developmental stages. The identification of such a large number of QTLs for seed weight and the differences in the number and location of QTLs in different studies including the QTLs that we have detected for seed weight and size in our present study, illustrate that seed weight and seed dimensions are complex traits which are controlled by many genetic loci. In addition, the interaction of these loci with the environment may also affect the identification, location and number of QTLs as shown with the different numbers and positions of the seedling QTLs under two different environmental conditions (Table 3).

There is experimental evidence that larger seeds are better able to establish or survive as seedling in a variety of environments, including nutrient shortage [24], [78]. This corroborates our observation of a greater correlation between seed weight and seedling vigor under nutrient-deprived condition than on MS medium with nutrients (Fig. 2). In general the shoot and root weights of the two parents as well as those in the RIL population were significantly lower under the nutrient-deprived conditions than those on vertical agar plates with MS nutrition. These results are in agreement with those reported by Nieuwhof *et al.* [68], who observed significant correlation between tomato seed size and seedling mass under nutrient-deprived conditions. We also observed some differences in the identification of QTLs between the two experiments. In general we identified higher numbers of QTLs with higher explained variance for three seedling traits (FrRtwn, DrRtwn FrShwn) in nutrient-deprived conditions (Table 3, Figure 3). For the nutrient deprived conditions, 9 out of 10 QTLs are overlapping with SW/SS QTLs, while for the growth of seedling with nutrients, 5 out of 7 seedling trait QTLs and 2 out of 3 HypL QTLs overlap with SW/SS QTLs. Although most seedling QTLs overlapped with seed dimension QTLs, we found some exceptions. A QTL for FrRt and HypL was found on chromosome 10 with explained variances from 8.5 and 9.3% respectively and another QTL on chromosome 12 for FrRt with an explained variance of 8.2%. Additionally a QTL for FrRtwn was found on chromosome 7 with an explained variance of 6.5%. The detection of these loci suggest the possibility for breeding for seedling vigour independent of seed size.

Genotype x environment interactions are very important for the expression of QTLs. In the present study identification of different QTLs in both of the environments indicates that some QTLs seem to be sensitive to the environment, but a substantial proportion of QTLs was found in both experiments. Especially the QTLs with higher LOD scores for all the traits could readily be detected in both environments. Therefore, the present study tends to support the general conclusion made by Tanksley [79], who concluded that a substantial proportion of QTLs affecting a trait can be identified under different environments, especially QTLs that have major effects.

Root systems execute the crucial task of providing water, nutrients and physical support to the plant. The length of the primary/main root and the number of the lateral roots determine the architecture of the root system. This root system in turn, plays a major role in determining whether a plant will succeed in a particular environment [34]. A fast-growing and improved deep root system will improve competitiveness with weeds during the initial stage of seedling growth. Furthermore it will also be more efficient in the acquisition of nutrients and uptake of water from lower layers of soil during low-nutrient- and low-moisture conditions. In soil or media with a patchy nutrient distribution, lateral roots preferentially proliferate in the nutrient-rich zone [80], [81] and thereby play an important role in the uniform utilization of nutrients from the soil. There are some studies which, in addition to its effect on the upward growth of seedlings, also demonstrate a correlation between seed traits (seed weight, -size and -vigor) on the initial downward growth of the root system [24], [82]. Finch-Savage *et al.* [35] found strong effects of seed vigor in *Brassica oleracea* on the initial downward growth of seedlings and fine mapped QTLs for rapid initial growth of root which also co-located with seed weight.

As the underground parts of plants are difficult to quantify, studies on roots are lagging behind those of shoots [64]. In the case of tomato no relevant information is available on root growth related traits nor has any proper study on seedling growth been published and, therefore, to the best of our knowledge, this is the first genetic analysis of seedling traits in tomato. Our results on root architecture tend to support the argument that larger food reserves in large-sized seed help in establishing an extensive root system. We observed that the heavy-weighted seed parent *S. lycopersicum* has a very strong root system with two times faster downwards growth (MRL = 8.54 cm) and two times bigger total root size (TRS = 13.99 cm) than the light-weighted seed parent *S. pimpinellifolium* with slow downward growth (MRL = 4.61 cm) and small total root size (TRS = 6.36 cm). These results are in agreement with the phenotypic values of fresh and dry root weights of the two parents. In total we identified 5 QTLs for MRL and 6 QTLs for TRS. For three major QTLs for MRL and for all the TRS QTLs, the positive alleles are derived from the *S. lycopersicum* parent (Table 3 and Fig. 3). In both of these cases, the major effect QTLs were also co-locating with SW and SS QTLs on linkage groups 9 and 11. On the other hand, the QTLs for LRn and LRD/Bz had 50% of the positive alleles from both parents with some major QTLs from the *S. lycopersicum* parent and these major QTLs were also co-locating with the seed size QTLs. The LRD/Bz value is relatively high for *S. pimpinellifolium*. This result illustrates that *S. pimpinellifolium* has a short branched zone with a high density of lateral roots, while *S. lycopersicum* has a longer branched zone with a lower density of lateral roots.

The co-location of QTLs for MRL, TRS, LRn, LRD/Bz and seed dimension traits with the positive additive effects from the same parent and the correlation of the phenotypic values for these traits, indicates that root and seed traits may be genetically interlinked traits and may be under the control of common genetic mechanisms.

For all the co-locations found in this study, it is not known whether it is a common allele controlling all the traits or whether it is a cluster of different alleles for different traits located closely together. Classical quantitative genetics assumes that trait correlation can be due to the effect of pleiotropy or due to the tight linkage of genes. For pleiotropic effects, one can expect not only the same location of QTLs for related traits, but also the same direction of their allelic effects. If close linkage of genes was the major reason, the directions of the genetic effects of the QTLs for different traits may be different, although coincidence of QTL locations can still be expected. The fact that most favorable alleles for the QTLs described in this study have been derived from the *S. lycopersicum* parent might suggest that pleiotropy rather than close linkage of different alleles is the major reason for correlation of the measured traits. In general, we found a high correlation between seed and seedling traits, but although we found co-localization of some RSA QTLs with seed dimension QTLs, the overall correlation between these traits was low. Eight out of the 17 RSA QTLs do not co-locate with seed dimension QTLs. These include major QTLs for LRn on chromosome 5 and for MRL on chromosome 7 with explained variances of 13 and 6.5% respectively and minor QTLs on chromosome 1 for MRL and TRS with explained variances of 6.1 and 5.6% respectively and on chromosome 3 and 10 for TRS explaining respectively 5.9 and 4.8% variance. These RSA QTLs together with the previous mentioned seeds size independent seedling weight QTLs indicate that in addition to seed size there are other mechanisms involved in controlling seedling establishment under different environmental conditions.

In conclusion, the strong co-location of QTLs among different seed and seedling traits with generally the same genetic direction of the QTLs and the correlation in the phenotypic values of these traits, indicate a strong correlation among seed- and seedling vigor and seed size and weight appear to have a strong effect on the initial downward growth of the main root and upward growth of the shoot. This positive effect of heavy seed could be due to common genetic mechanisms controlling these traits and also to the high quantity of reserve food in larger seeds as compared to small seeds.

Apart from the correlation between seed and seedling traits we also tested the correlation between seed weight and seed performance in a previous analysis [53], but found no significant correlation between seed weight and germination rate (t_50_), uniformity (U_7525_) and final germination percentage (G_max_%). Thus, increased seed size seems a benefit for seedling establishment, but a consistent link between seed size and germination characteristics is not obvious. In tomato it has been reported that inheritance of time to germination was closely related to seed size, with the smaller seeds germinating earlier [83]. However, our data show that this is not the case for the here studied population. Furthermore we have also shown that germination performance and seed size are controlled by different independent genetic loci [53].

The mapping of QTLs associated with key seed- and seedling-vigour traits in tomato could open up various opportunities to improve efficiency of plant breeding and selection for lines with improved seed vigor and, hence, seedling and crop establishment. Molecular markers linked to the QTLs may be utilized in marker-assisted selection, providing a rapid method to select for specific genotypes without the need to extensively assess phenotypes at all stages in the breeding program. Furthermore, we will follow up the defined QTLs with fine-mapping and improvement of candidate-gene selection by the use of a genetical genomics set-up and thereby elucidate the molecular mechanisms that control seed- and seedling-vigour [84], [85].
